# Supplementary material for: Aromatisation of steroids in the bivalve Mytilus trossulus
Source: PeerJ. 2019 May 22;7:e6953. doi: 10.7717/peerj.6953 (PMC6535040; doi:10.7717/peerj.6953)
Supplement: Supplemental Information 1 — Data presented as mean ± SD (n = 4). [file peerj-07-6953-s001.docx]

| Microsomes isolated from  gills and gonads without sexing | AE (pmol/h/mg protein) ± SD | |
| --- | --- | --- |
|  | gills | gonads |
| A. incubation with NADPH | 61.98 ± 13.78 | 78.14 ± 17.71 |
| A. incubation without NADPH | 6.40 ± 0.13 | 14.70 ± 0.04 |
| B. "active" microsomes | 29.69 ± 7.80 | 31.16 ± 8.25 |
| B. denatured microsomes | 4.99 ± 0.32 | 9.83 ± 0.42 |
