# Supplementary material for: Aromatisation of steroids in the bivalve Mytilus trossulus
Source: PeerJ. 2019 May 22;7:e6953. doi: 10.7717/peerj.6953 (PMC6535040; doi:10.7717/peerj.6953)
Supplement: Supplemental Information 2 — F, females; M, males; ND, steroids labeled with 13C3 not detected. [file peerj-07-6953-s002.docx]

A)

| Ind. no. | Androgen-^13^C_3_ exposure  (in acetonitrile): | Wet weight (grams) | A-^13^C_3_ (ng/g w. w.) | T-^13^C_3_ (ng/g w.w.) | E1-^13^C_3_ (ng/g  w. w.) | 17βE2-^13^C_3_ (ng/g w. w.) |
| --- | --- | --- | --- | --- | --- | --- |
| 1 | Acetonitrile (control) | 0.6 | ND | ND | ND | ND |
| 2 | Acetonitrile (control) | 0.9 | ND | ND | ND | ND |
| 3 | Acetonitrile (control) | 1.5 | ND | ND | ND | ND |
| 4 | Acetonitrile (control) | 1.6 | ND | ND | ND | ND |
| 5 | Acetonitrile (control) | 1.3 | ND | ND | ND | ND |
| 6 | 4-Androstene-3,17-dione-2,3,4-^13^C_3_ | 1.6 | 2.02 | 1.30 | 1.43 | 0.48 |
| 7 | 4-Androstene-3,17-dione-2,3,4-^13^C_3_ | 1.6 | 1.39 | 1.29 | ND | ND |
| 8 | 4-Androstene-3,17-dione-2,3,4-^13^C_3_ | 1.5 | 2.38 | 1.59 | 1.49 | 0.52 |
| 9 | 4-Androstene-3,17-dione-2,3,4-^13^C_3_ | 1.0 | ND | ND | ND | ND |
| 10 | 4-Androstene-3,17-dione-2,3,4-^13^C_3_ | 1.1 | 3.27 | 1.17 | 1.15 | 0.60 |
| 11 | Testosterone-2,3,4-^13^C_3_ | 0.9 | 1.17 | **10.51** | 0.54 | ND |
| 12 | Testosterone-2,3,4-^13^C_3_ | 1.3 | 1.15 | **6.86** | 0.34 | 0.96 |
| 13 | Testosterone-2,3,4-^13^C_3_ | 0.9 | 0.87 | **7.37** | 0.42 | 1.46 |
| 14 | Testosterone-2,3,4-^13^C_3_ | 1.1 | ND | ND | ND | ND |
| 15 | Testosterone-2,3,4-^13^C_3_ | 1.8 | 0.84 | **4.94** | 0.44 | 0.65 |

B)

| Ind. no./  sex | Androgen-^13^C_3_ exposure (in acetonitrile): | Wet weight (grams) | A-^13^C_3_ (ng/g w. w.) | T-^13^C_3_ (ng/g  w.w.) | E1-^13^C_3_ (ng/g  w. w.) | 17βE2-^13^C_3_ (ng/g w. w.) |
| --- | --- | --- | --- | --- | --- | --- |
| 16/F | 4-Androstene-3,17-dione-2,3,4-^13^C_3_ | 0.86 | 1.23 | 2.11 | 3.50 | ND |
| 17/F | 4-Androstene-3,17-dione-2,3,4-^13^C_3_ | 0.73 | 4.51 | 2.73 | 4.42 | 0.91 |
| 18/M | 4-Androstene-3,17-dione-2,3,4-^13^C_3_ | 0.84 | 3.00 | 2.81 | 3.84 | 0.64 |
| 19/F | 4-Androstene-3,17-dione-2,3,4-^13^C_3_ | 0.67 | 5.39 | 2.02 | 4.44 | 0.87 |
| 20/M | 4-Androstene-3,17-dione-2,3,4-^13^C_3_ | 0.53 | 5.33 | 2.11 | 4.46 | 0.70 |
| 21/M | Testosterone-2,3,4-^13^C_3_ | 1.23 | 0.70 | **8.11** | 0.48 | 1.05 |
| 22/M | Testosterone-2,3,4-^13^C_3_ | 1.08 | 0.59 | **8.63** | ND | 1.25 |
| 23/M | Testosterone-2,3,4-^13^C_3_ | 0.52 | 1.53 | **12.76** | 0.36 | 1.34 |
| 24/M | Testosterone-2,3,4-^13^C_3_ | 0.33 | ND | **13.47** | 0.73 | 1.13 |
| 25/F | Testosterone-2,3,4-^13^C_3_ | 0.66 | 0.87 | **3.69** | 0.87 | 0.87 |
